# Intimate partner violence and associated factors among pregnant women in Ethiopia: a systematic review and meta-analysis

**DOI:** 10.1186/s12978-018-0637-x

**Published:** 2018-12-04

**Authors:** Animut Alebel, Getiye Dejenu Kibret, Fasil Wagnew, Cheru Tesema, Aster Ferede, Pammla Petrucka, Firew Tekle Bobo, Molla Yigzaw Birhanu, Animen Ayehu Tadesse, Setegn Eshetie

**Affiliations:** 1 College of Health Sciences, Debre Markos University, P.O. Box 269, Debre Markos, Ethiopia; 2 College of Nursing, University of Saskatchewan, Saskatoon, Canada; 3 School of Life Sciences and Bioengineering, Nelson Mandela African Institute of Science and Technology, Arusha, Tanzania; 4 Department of Public Health, Wollega University, Nekemte, Ethiopia; 5 Felege Hiwot Referral Hospital, Bahir Dar, Ethiopia; 6 College of Medicine and Health Sciences, University of Gondar, Gondar, Ethiopia

**Keywords:** Prevalence, Intimate partner violence, Meta-analysis, Systematic review, Ethiopia

## Abstract

**Background:**

Intimate Partner Violence (IPV) during pregnancy is a serious public health concern globally. Within Sub-Saharan Africa nearly 40% of women reported abuse by their intimate partners. In Ethiopia, study findings regarding prevalence and associated factors of IPV among pregnant women have been inconsistently reported and highly variable. Thus, this systematic review and meta-analysis estimates the pooled prevalence of IPV and associated factors among pregnant women in Ethiopia.

**Methods:**

International databases (i.e., PubMed, EMBASE, CINAHL, Google Scholar, Science Direct, and the Cochrane Library) were systematically searched during the period of January 1 to February 13, 2018. All identified observational studies reporting the prevalence of IPV and associated factors among pregnant women in Ethiopia were considered. Two authors (AA and CT) independently extracted all necessary data using a standardized data extraction format. Extracted quantitative data were analyzed using STATA Version 13. Heterogeneity among the included studies was assessed through the Cochrane Q test statistics and *I*^*2*^ test. Finally, a random effects meta-analysis model was computed to estimate the pooled prevalence of IPV. Associations between factors and IPV were also examined using a random effects model.

**Results:**

After reviewing 605 studies, eight studies involving 2691 pregnant women fulfilled the inclusion criteria and were included in this meta-analysis. The findings of these eight studies revealed that a 26.1% (95% CI: 20, 32.3) overall prevalence of IPV among pregnant women in Ethiopia. The subgroup analysis of this study further revealed the highest observed prevalence was in Oromia region (35%), followed by Amhara region (29%). Mothers‘educational status (OR: 2.1, 95% CI: 1.1, 3.7), intimate partners’ educational status (OR: 3.5, 95%CI: 1.4, 8.5), and intimate partners’ alcohol use (OR: 11.4, 95%CI: 2.3, 56.6) were significantly associated with IPV among pregnant women.

**Conclusion:**

This study found that the prevalence of IPV among pregnant women in Ethiopia was quite common; with slightly more than 1 in 4, pregnant women experienced IPV during pregnancy. Mothers’ educational status, intimate partners’ educational status, and intimate partners’ alcohol use were factors significantly associated with IPV among pregnant women.

**Electronic supplementary material:**

The online version of this article (10.1186/s12978-018-0637-x) contains supplementary material, which is available to authorized users.

## Plain English summary

IPV during pregnancy is a serious public health concern throughout the world. In Ethiopia, study findings regarding prevalence and associated factors of IPV amongst pregnant women have been inconsistent and highly variable. Therefore, this systematic review and meta-analysis estimates the pooled prevalence of IPV and associated factors among pregnant women in Ethiopia.

International databases (i.e., PubMed, EMBASE, CINAHL, Google Scholar, Science Direct, and the Cochrane Library) were systematically searched. A total eight studies involving 2, 691 pregnant women fulfilled the inclusion criteria and were included in the meta-analysis. The two authors independently extracted all necessary data using a standardized data extraction format. Data analysis was carried out using STATA Version 13 statistical software. The Cochrane Q test statistics and *I*^*2*^ test were used to assess heterogeneity of the included studies. Finally, a random effects meta-analysis model was computed to estimate the pooled prevalence of IPV. Moreover, the associations between factors and IPV were also examined using the random effects model.

In this study, the prevalence of IPV in Ethiopia showed slightly more than 1 in 4pregnant women experienced IPV during pregnancy. Both partners’ educational status and the intimate partners’ alcohol use were factors significantly associated with IPV among pregnant women in Ethiopia.

## Background

Intimate Partner Violence (IPV) includes physical, sexual, and emotional abuse against women by an intimate partner [1, 2]. It is a serious public health concern throughout the world, but it’s notably present in Sub-Saharan Africa, where 38.83% of the women abused by their intimate partner [3, 4]. IPV is a common phenomenon in both urban and rural families of Ethiopia. In a multi-country based study conducted by World Health Organization (WHO) on women’s health and domestic violence against women found that the prevalence of IPV during pregnancy in Ethiopia was estimated to be 8% [5].

Violence during pregnancy has devastating health and social consequences, both for the woman and for the developing fetus [1, 6, 7]. Unintended pregnancies, pregnancy-related symptom distress (antenatal, intranatal and postnatal depression), inadequate prenatal care, induced abortion, spontaneous abortion, gestational weight gain, intrauterine restriction, hypertension, pre-eclampsia, third trimester bleeding, and STIs (HIV and others) were the most frequent adverse maternal health related outcomes after IPV during pregnancy [8, 9]. Unless interventions are undertaken, IPV during pregnancy can result in a deleterious effect on birth outcomes, ranging from the adverse outcomes above to preterm birth to fetal and/or maternal death [10–12]. Therefore, early identification and intervention of IPV during pregnancy is necessary in reducing such adverse and preventable outcomes [13].

In Ethiopia, studies assessing IPV and associated factors among pregnant women have contributed a range of findings [14–21]. These small and fragmented studies demonstrated that IPV during pregnancy ranged from 12% in Yirgalem town, Southern Nations, Nationalities, and Peoples Region (SNNPR) [16] to 44.5%in Abay Chomen District, Oromia Region [21]. Such discrepancy has not been yet investigated. Regarding associated factors, previous studies have cited mothers’ educational status [14–17, 19, 20], intimate partners’ educational status [14, 15, 17, 21], unplanned pregnancy [15, 16, 19], and intimate partners’ alcohol use [15, 17, 20] as the common factors contributing to IPV among pregnant women in Ethiopia.

There was no a nationwide study assessing the magnitude and associated factors of IPV among pregnant. Thus, this systematic review and meta-analysis aims to estimate the pooled prevalence and associated factors of IPV among pregnant women in Ethiopia using available studies. The findings of this systematic review and meta-analysis will highlight the prevalence and associated factors of IPV with implications to improve health workers’ interventions, ensure cost-effectiveness, and accelerate the reduction of IPV among pregnant women.

## Methods

### Search strategies

This meta-analysis was prepared and presented according to the Preferred Reporting Items for Systematic Reviews and Meta-Analysis (PRISMA) [22] (see Additional file 1). To find potentially relevant articles, a comprehensive search with no date limit was performed in the following databases: PubMed/MEDLINE, EMBASE, CINAHL, Google Scholar, Science Direct and Cochrane Library. All searches were limited to articles written in English which does not alter the outcome of the systematic reviews and meta-analyses [23]. Grey literature was searched through the review of reference lists and input of content experts. Moreover, to find unpublished papers in the field of our systematic review and meta-analysis, some research centers, including the Addis Ababa Digital Library were searched. Studies identified by our search strategy were retrieved and managed using Endnote X7 (Thomson Reuters, Philadelphia, PA, USA) software. The search was conducted between January 1 and February 13, 2018. All papers published until February 13, 2018 were included. The search used the following keywords “prevalence”, “intimate partner violence among pregnant”, “domestic violence among pregnant”, “associated factors”, “determinants”, and “Ethiopia”. The search terms were used separately and in combination using Boolean operators like “OR” or “AND”.

### Inclusion and exclusion criteria

#### Inclusion criteria

**Study area**: Only studies conducted in Ethiopia.

**Population:** Only studies involving pregnant women.

**Publication condition:** Both published and unpublished articles were included.

**Study design:** All observational study designs (i.e., cross-sectional, case-control and cohort) reporting the prevalence of IPV were eligible for this meta-analysis.

**Language:** Only studies reported in the English language were considered.

### Exclusion criteria

Before exclusion, we examined the eligibility of the studies after reading their titles and abstracts. Then, if the studies were thought relevant to our review, we examined the full texts. During the article selection process, studies, which were not fully accessible (full text available), were excluded. However, before excluding the articles, we attempted to contact the primary author at least two times through email. The reason for the exclusion of these articles is our inability to assess the quality of each article in the absence of their full texts. In addition, studies not reporting our outcome of interest were excluded after reviewing their full texts.

### Outcome measurements

This review considered two main outcomes. IPV during pregnancy, as the primary outcome variable of this study, is defined as emotional, physical, or sexual abuse, or stalking that occurs among individuals in an intimate (close) relationship including current and former spouses and dating partners [24]. Psychological violence was defined as any form of insults included insult, humiliation, intimidates on purpose, threatened to hurt women or someone she cared about [15].The second outcome of this study was to identify factors associated with IPV among pregnant women. For the second outcome, we determined the association between IPV and associated factors in the form of the log odds ratio. Four major factors assessed by each primary studies were selected to explore their association with intimate partner violence. For each factor, the odds ratio was calculated based on the binary outcome data reported by each study. The factors assessed for this review were educational status of mothers (unable to read and write versus able to read and write), educational status of the intimate partner (unable to read and write versus able to read and write), intimate partner alcohol use (yes versus no), and unplanned pregnancy (yes versus no).

### About WHO tool used by primary studies

Almost all research included in this meta-analysis used the WHO tool for the assessment of IPV. One of the main challenges facing during the WHO multi-centered study was to develop clear definitions of different types of violence that permit meaningful comparisons among diverse settings. The tool was developed after a long process of discussion and consultation. It was translated and pretested in six countries (Bangladesh, Brazil, Namibia, Samoa, Thailand, and the United Republic of Tanzania) and retested in the remaining participating countries [25]. This multi-center study also included Ethiopia.

### Data extraction

Two authors (AA and CT) independently extracted all necessary data using a standardized data extraction form, which was adapted from the JBI data extraction format. At the time of data collection, any disagreements between the two authors were resolved through discussion and consensus (i.e., a Delphi process). If additional information or clarification was needed, the primary author of the original research was contacted. For the first outcome (prevalence), the data extraction form included primary author, publication year, regions of the country where the study was conducted, study area, sample size, study design, response rate and prevalence with 95% confidence intervals. For the secondary outcome (associated factors), data were extracted in a format of two by two tables, and then the log odds ratio for each factor was calculated based on the reports of original studies.

### Quality assessment

Two authors (AA and CT) independently assessed the quality of each original study using the quality assessment tool. To assess the quality of studies, the Newcastle-Ottawa Scale for cross-sectional, cohort, and case control studies quality assessment was adapted and used [26]. This tool has three main sections. The first section scored on the basis of one to five stars focuses on the methodological quality of each study (i.e., sample size, response rate, and sampling technique). The second section of the tool considers the comparability of the study cases or cohorts with a possibility of two stars to be gained. The last section is concerned with the outcomes and statistical analysis of the original study with a possibility of three stars to be gained. Any disagreements between the two reviewers were resolved by taking the mean score of the two reviewers’. Finally, articles assessed with a score of ≥6 out of 10 were considered as achieving high quality. This cut-off point was declared after reviewing relevant literature.

### Data processing and analysis

Before analysis, necessary data from each original study were extracted using Microsoft Excel spreadsheet form. The data were imported into STATA Version 13 statistical software for further analysis. The standard error for each original study was calculated using the binomial distribution formula. Heterogeneity among reported prevalence was assessed by computing *p*-values for chi-square test, Q-statistics, and*I*^2^ test [27]. Based on the results of the statistical test, significant heterogeneity was exhibited among the included studies (*I*^2^ = 93.0%, *p* < 0.001), hence, a random effects meta-analysis model was computed to estimate the Der Simonian and Laird’s pooled effect. To minimize the random variations between the point estimates of the primary study subgroup, analysis was done based on study settings (i.e., region where the study were conducted, residence, educational status of mothers and intimate partners, intimate partner alcohol use, and unplanned pregnancy). Moreover, to identify the possible source of heterogeneity, we performed univariate meta-regression by considering sample size, region of the country, and year of publication as covariates; however, none were found to be statistically significant. Egger’s and Begg’s tests at a 5% significance level were not significant for publication bias [28]. Point prevalence, as well as 95% confidence intervals, was presented in a forest plot format. In this plot, the size of each box indicated the weight of the study, while each crossed line referred to 95% confidence interval. For the secondary outcomes, a log odds ratio was used to determine the association between IPV and associated factors.

## Results

**Fig. 1:**
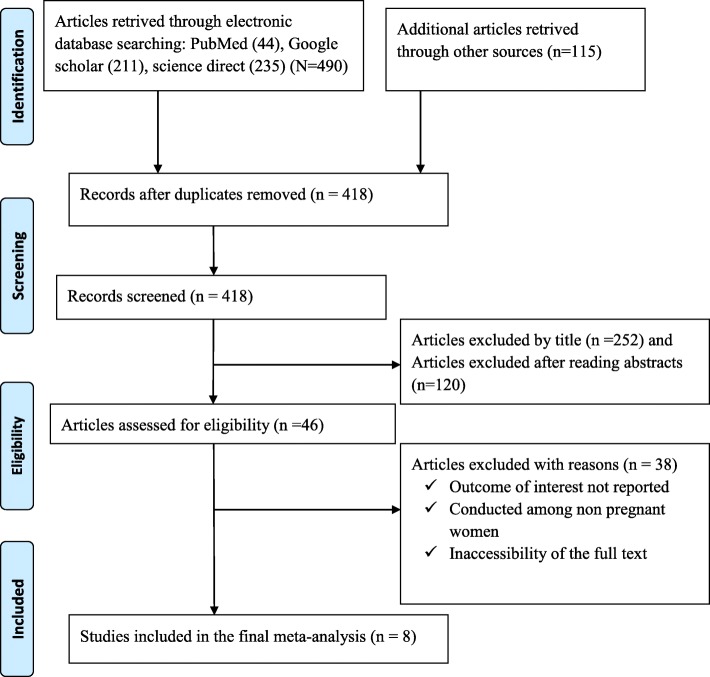
Flow chart of study selection for systematic review and meta-analysis of intimate partner violence and associated factors among pregnant women in Ethiopia

In the first step of our search, 605 potentially relevant articles regarding IPV and associated factors among pregnant women were collected from PubMed/MEDLINE, EMBAS, CINAHL, Google Scholar, Science Direct, Cochrane Library, and other sources described previously. Of these initial articles, 187 articles were excluded due to duplications. Of the remaining 418 articles, 372 articles were excluded after review of their titles and abstracts as non-relevant to this review. Therefore, 46 Articles were accessed, and assessed for eligibility based on the pre-set criteria, further yielding exclusion of 38 articles primarily due to the outcome of interest and study population [13, 29–41]. Eight (8) studies met the inclusion criteria and were considered in the final meta-analysis (Fig. 1).

### Characteristics of original studies

As shown below in Table 1, in the present meta-analysis 2756 pregnant women participants were identified of whom, 2691were involved in the studies, yielding a response rate of 97.6%. From the reports of primary studies, the mean age of the respondents ranged from 25±6 [16] to 29.8±5.8 [14] years. Concerning publication year and study design, most articles (87.5%) were cross sectional, and published between 2014 to 2017 respectively. The sample size of the studies ranged from 195 to 434. In this review, the lowest prevalence (12%) of IPV was found in a study conducted at the Yirgalem Health Center, SNNPR [16], while the highest prevalence (44.7%) of IPV was reported in a study conducted at Abay Chomen District, Oromia Region [21]. These eight studies were conducted in four Ethiopian regions and in one administrative town. Two of each studies were conducted in Amhara [14, 19], Oromia [18, 21] and SNNPR regions [15, 16], whilst one each occurred in Tigray [17] and Addis Ababa [20]. No studies were reported from Benishangul Gumiz, Harari, Afar, Somali and Gagmbela regions. Quality scores of each original study ranged from a low of three to a high of eight. In relation to response rate, almost all studies had a good response rate (> 85%), which may, in part, be attributable to the use of interviewer-administered questionnaires to collect the data (Table 1).

**Table 1:** Descriptive summary of eight studies included in the meta-analysis of IPV and associated among pregnant women in Ethiopia factors of 2018

Author	Publication year	Region	Study Area	Study Design	Sample size	Response rate	Prevalence (%)
Kassa and Menale [16]	2016	SNNPR	Yirgalem Health Center	Facility based cross sectional	216	100	12
Abate et al. [21]	2016	Oromia	Abay Chomen District	Community based cross sectional	299	94.3	44.7
Gebrezgi et al. [17]	2017	Tigray	Shire Endaselassie Town	Facility based cross-sectional	422	100	20.6
Yimer et al. [14]	2014	Amhara	Hulet Ejju Enessie District	Community based cross-sectional	434	97.9	32.2
Laelago et al. [15]	2014	SNNPR	Hossana Town	Facility based cross sectional	195	94	19.7
Demelash et al. [18]	2015	Oromia	Goba, Robe, Delomena, and Ginir	A hospital-based case control	408	94	25.8
Bifftu et al. [19]	2017	Amhara	Gondar Town	A clinical based cross-sectional	422	99.1	25.4
Abdurashid and Tesfahun [20]	2013	Addis Ababa	Addis Ababa	Facility based cross sectional	360	96.7	29.3

### Meta-analysis

**Fig. 2:**
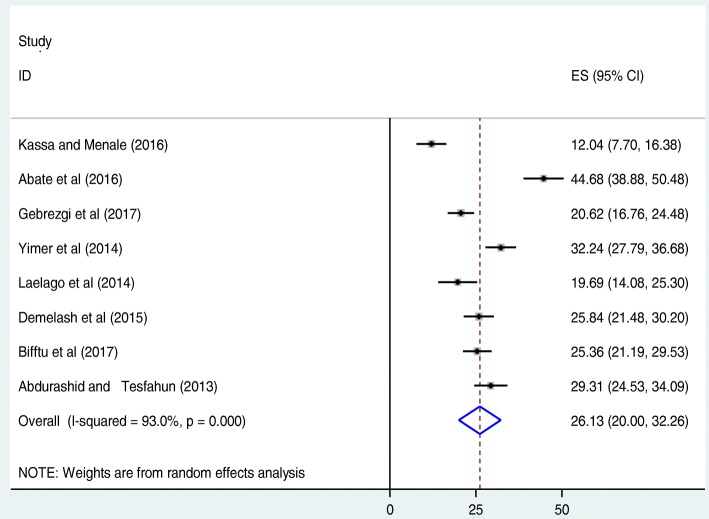
Forest plot of the pooled prevalence of IPV among pregnant women in Ethiopia

The overall prevalence calculated from the eight included Ethiopian studies showed a pooled prevalence of IPV during pregnancy was found to be 26.1% (95%CI: 20, 32.3) (Fig. 2). The included studies exhibited significant heterogeneity (I^2^ = 93.0, *p* < 0.001), which led us to compute a random effect meta-analysis model to estimate the pooled prevalence of IPV during pregnancy in Ethiopia. To identify the possible sources of heterogeneity, different factors associated with the heterogeneity such as year of publication, sample size, and region of the country where the study conducted, were investigated using univariate meta-regression models, but none of these variables were found to be statistically significant (Table 2). Publication bias was also assessed using Begg’s and Egger’s tests, which showed no statistical significance for estimating the prevalence of IPV among pregnant women in Ethiopia with *p*-value of *p* = 0.08 and *p* = 0.2 respectively.

**Table 2:** Related factors with heterogeneity of IPV among pregnant women in the current meta-analysis (based on univariate meta-regression model)

Variables	Coefficient	*P*-value
Publication year	−2.1	0.7
Sample size	0.1	0.5
Regions		
Amhara	13.0	0.2
Oromia	19.2.53	0.08
Others	9.1	0.3
SNNPR (reference)	0	

### Subgroup analysis

In this meta-analysis, we performed a multiple comparison of the prevalence of IPV among pregnant women by taking different factors. The factors included region of the country, types of violence, residence, woman’s educational level, intimate partner’s educational level, intimate partner’s alcohol use, and planned/unplanned pregnancy. Regarding regional prevalence, the highest IPV was observed in Oromia Region with a prevalence of 35% (95%CI: 17, 54) followed by Amhara at 29% (95%CI: 22, 36), and then others (Tigray and Addis Ababa) at 25% (95%CI: 16, 34). Likewise, the subgroup analysis of this study indicated that the most common form of violence was psychological violence with a prevalence of 21% (95%CI: 9, 33) followed by physical violence with a prevalence of 16% (95CI: 12, 21). Mothers who are unable to read and write were experiencing more IPV as compared to their counterparts. In addition, a woman whose intimate partner is unable to read and write encountered IPV almost twice as much as compared to their counterparts. Moreover, women with unplanned pregnancy experienced higher IPV as compared to women with planned pregnancy. Furthermore, rural mothers faced higher IPV at the time of pregnancy compared to urban counterparts (Table 3).

**Table 3:** Subgroup prevalence of intimate partner violence among pregnant women in Ethiopia, 2018 (*n* = 8)

Variables	Subgroup	No. of studies	Event	N	Prevalence (95%CI)	I^2^ (%)	*P*-value
Types of violence	Physical	7	380	2304	16 (12, 21)	90.8	< 0.001
	Psychological	6	429	2088	21 (9, 33)	98.4	< 0.001
	Sexual	5	198	1666	12 (5, 20)	96.3	< 0.001
Regions of the country	SNNPR	2	64	409	16 (8, 23)	77.6	0.03
	Oromia	2	226	669	35 (17, 54)	96.1	< 0.001
	Amhara	2	243	843	29 (22, 36)	79.6	0.03
	Others	2	189	770	25 (16, 34)	87.0	0.006
Maternal educational level	Unable to read and write	6	328	940	34 (31, 38)	0	0.76
	Able to read and write	6	249	1148	22 (14, 30)	91.43	< 0.001
Intimate partner educational level	Unable to read and write	4	185	375	50 (45, 55)	0	0.68
	Able to read and write	4	206	886	25 (11, 39)	96.3	< 0.001
Unplanned Pregnancy	Yes	3	166	562	11 (39, 89)	99.7	< 0.001
	No	3	275	547	53 (44, 62)	67.7	0.05
Intimate partner alcohol use	Yes	3	177	357	54 (37, 72)	90.9	< 0.001
	No	3	65	580	11 (3, 11)	89.8	< 0.001
Residence	Rural	2	86	169	51 (43, 58)	0	–
	Urban	2	107	671	15 (12, 17)	0	–

### Factors associated with intimate partner violence

#### Association between mother’s educational status and IPV

**Fig. 3:**
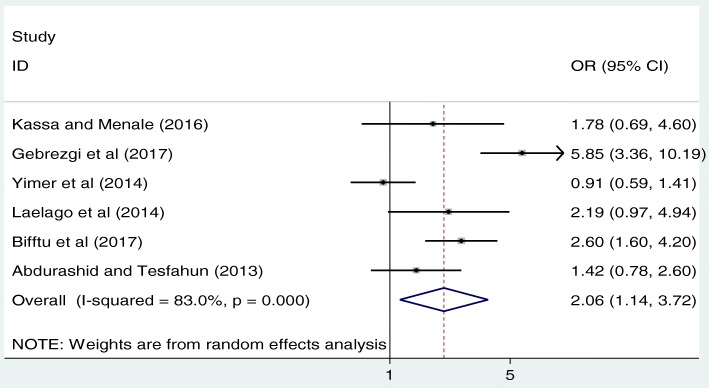
The pooled odds ratio of the association between maternal education and IPV in Ethiopia

As shown below in Fig. 3, we examined the association between mothers’ educational status and IPV based on the reports of six studies [14–17, 19, 20]. The result of these six studies indicated that mother’s educational status was significantly associated with IPV. The pooled odds ratio indicated that the likelihood of IPV occurrence was 2.1 times higher among mothers’ who were unable to read and write as compared to their literate counterparts (OR: 2.1, 95% CI: 1.1, 3.7). In this analysis, high heterogeneity (I^2^ = 83.0% and *p* < 0.001) was exhibited; as a result, a random effect meta-analysis model was computed to determine the association. Besides, publication bias was assessed using Begg’s and Egger’s tests. The result of these tests revealed that there was a low possibility of publication bias with *p*-value of 0.85 and 0.9 respectively (Fig. 3).

#### The association between unplanned pregnancy and IPV

**Fig. 4:**
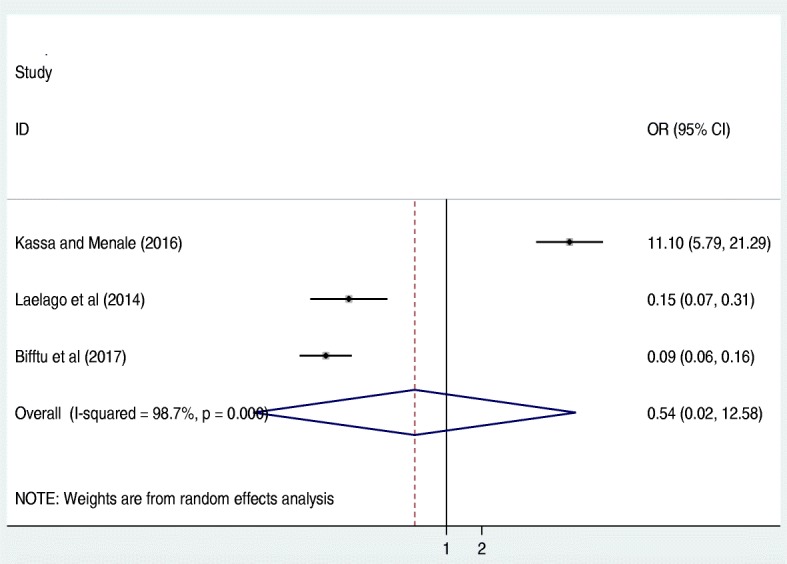
The pooled odds ratio of the association between unplanned pregnancy and IPV in Ethiopia

From the meta-analysis of three studies [15, 16, 19], unplanned pregnancy was not significantly associated with IPV (OR: 0.5, 95% CI: 0.02, 12.58). The included studies exhibited severe heterogeneity (I^2^ = 98.7% and *p* < 0.001) as a result, a random effect meta-analysis was employed to do the final analysis. Publication bias assessed by using Begg’s and Egger’s tests revealed that there was no possibility of publication bias with *p*-value of 0.90 and 0.42 respectively (Fig. 4).

#### Association between intimate partner’s education and IPV

**Fig. 5:**
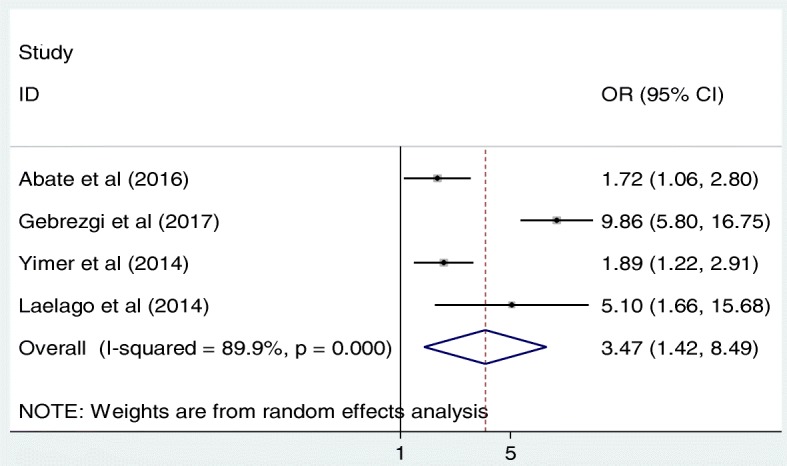
The pooled odds ratio of the association between intimate partner educational status and IPV in Ethiopia

In addition, to determine the association between the level of education of the intimate partner and IPV, four studies were included in the analysis [14, 15, 17, 21]. The pooled odds ratio of this meta-analysis indicated that pregnant women whose intimate partner is unable to read and write were 3.5 times more likely to experience IPV as compared to those literate counterparts (OR: 3.5, 95%CI: 1.4, 8.5) (Fig. 5). In this meta-analysis, the included studies were characterized by high heterogeneity (I^2^ = 89.9%; *p* < 0.001) resulting in use of a random effect meta-analysis model. Publication bias was also assessed by using Begg’s and Egger’s tests with *p*-values of 0.50 and 0.63 respectively.

#### The association between intimate partner alcohol use and IPV

**Fig. 6:**
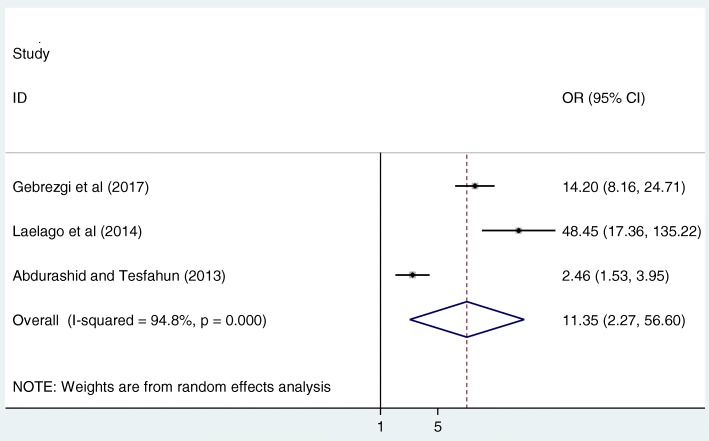
The pooled odds ratio of the association between intimate partner alcohol use and IPV in Ethiopia

Finally, in this meta-analysis, we assessed the association between intimate partner alcohol use and IPV. The overall pooled result of this study revealed that pregnant women whose intimate partners consumed alcohol were 11.4 times more likely to be abused as compared to their counterparts (OR: 11.4, 95%CI: 2.3, 56.6) (Fig. 6). High heterogeneity (I^2^ = 94.8%; *p*-value< 0.001) was observed among the included studies; hence, a random effect meta-analysis model was employed to estimate the final analysis. Furthermore, the Begg’s and Egger’s tests indicated that there was low publication bias with *p*-values of 0.12 and 0.40 respectively.

## Discussion

This meta-analysis was conducted to estimate the national prevalence of IPV during pregnancy and associated factors. To the best of our knowledge, this meta-analysis is the first of its kind to estimate the pooled prevalence of IPV and associated factors among pregnant women in Ethiopia. This review indicated a wide range of pregnancy-related IPV prevalence rates, ranging from as low as 12.0% to as high as 44.7%. This wide range prevalence is comparable with systematic review conducted in other African countries(2–57%) [3]. The pooled prevalence of IPV during pregnancy in Ethiopia was 26.1% (95%CI: 20, 32.3). The finding is in agreement with a systematic review conducted in Nigeria, which showed the prevalence of IPV during pregnancy ranged between 2.3 and 44.6% [42]. However, this finding is much higher than a meta-analysis conducted in China, which found the prevalence of IPV among pregnant women as 7.7% [43]. Our finding is also much higher than a meta-analysis conducted among pregnant women in African countries, which estimated the prevalence of IPV among pregnant women as 15.23% [3].

The difference in prevalence rates could be explained by the differences in community awareness regarding IPV during pregnancy. Another possible explanation for the variation may be due to the differences in educational level, accessibility of information on gender-based issues, reproductive health information, geographical areas, and the cultures of study subjects. Moreover, the difference in the prevalence of IPV between this meta-analysis and a meta-analysis conducted in China could be due to the difference tools used by individual studies conducted in each country. For example, three studies conducted in China used the Chinese version of the Abuse Assessment Screen questionnaire as the evaluation tool [44–46]. Besides, there is cultural difference between Chinese and Ethiopian women. A study done by Rachael S at the University of Michigan indicated that Ethiopian women had high acceptance rate of intimate partner violence [47]. Therefore, this could be another possible explanation for the difference of IPV between China and Ethiopia.

The subgroup analysis of this study indicated that the highest prevalence of IPV was observed in Oromia region, 35% (95%CI: 17, 54) followed by Amhara region, 29% (95%CI: 22, 36) whereas the lowest prevalence was observed in SNNPR with a prevalence of 16% (95%CI: 8, 2). A possible explanation for this variation could be due to the differences in community perceptions towards IPV. Additionally, there could be a difference in study design, as studies conducted in Oromia and Amhara regions included both community-based and facility-based studies whereas studies conducted elsewhere included only facility-based studies. Therefore, facility-based studies could miss t0hose women who were not coming for requiring formal health services. In addition, this meta-analysis disclosed that the prevalence of psychological violence (21%) was higher than physical (16%) and sexual violence (12%). Our finding is in line with a meta-analysis conducted in China, which revealed that the prevalence of psychological, physical, and sexual violence were 4.2, 3.6, and 1.3% respectively [43].

In this meta-analysis, we also explored factors associated with IPV among Ethiopian pregnant women. The results indicated that only the variables woman’s and her intimate partner’s educational status, and intimate partner’s alcohol consumption were significantly associated with IPV during pregnancy. Accordingly, the likelihood of IPV occurrence was 2.1 times higher among mothers’ who were unable to read and write as compared to their literate counterparts. This finding is consistent with studies from Nigeria, Tanzania, and Rwanda, which found that pregnant women with no formal education were more likely to experience violence by their intimate partners [48–50]. This finding could potentially be attributed to these women having less access to information concerning women empowerment or more acceptances for IPV compared to literate counterparts [51].

The present meta-analysis showed that the intimate partners’ education was significantly associated with IPV. Our findings found that pregnant women with an intimate partner that is unable to read and write, were 3.5 times more likely to experienced IPV as compared to those who had an intimate partner able to read and write. This finding aligns with studies conducted in Kenya and Bangladesh, which found that having a partner attending tertiary education is a protective factor against IPV during pregnancy, and intimate partner’s education beyond 10^th^grade was, in both rural and urban areas, significantly associated with lower odds of IPV during pregnancy [52, 53]. This finding may reflect that intimate partners with no formal education will probably have traditional perceptions with respect to gender equality [52]. On the contrary, a study conducted in Nigeria indicated that women who had intimate partners with no formal education were lower risk of experiencing IPV [50].

Furthermore, in this meta-analysis, we observed that the intimate partner’s alcohol intake was significantly associated with IPV. Pregnant women whose intimate partners consumed alcohol were 11.4 more likely to be abused as compared to their non-alcohol consumption counterparts. This finding is in line with a systematic review and meta-analysis conducted in China [43]. Study findings from this review suggested that a woman who had an occasional alcohol-drinking partner and women who had a heavy alcohol-drinking partner were more likely to experience IPV than compared to women whose partner did not drink during pregnancy. This finding could be explained by alcohol’s influence on psychological and physical capacities, as well as relationship dynamics, which could prompt a decline in a couple’s capacity to solve rather than escalate conflicts. Another possible explanation may relate to the increased financial burden on the entire family due to alcohol [54, 55].

### Limitations of the study

This meta-analysis was considered only articles or reports conducted in the English language, which may have restricted some papers from being included. In addition, the majority (87.5%) of included studies were cross-sectional in nature; as a result, the outcome variables might be affected by other confounding variables. The majority of the studies included in this review had a relatively small sample size, which could affect the estimated prevalence reported. Although almost all research included in our meta-analysis used the WHO tool for the assessment of IPV, the occurrence of IPV was determined based on the reports of women, which might be affected by social desirability bias. Furthermore, this meta-analysis represented only studies reported from four regions and one administrative town of the country, which may yield an under-representation of prevalence.

## Conclusion

This meta-analysis found that the prevalence of IPV among pregnant women in Ethiopia was significant with slightly more than 1 in 4 pregnant women experiencing IPV during pregnancy. Mothers’ and intimate partners’ educational status, as well as the intimate partners’ alcohol use were factors significantly associates with IPV among pregnant women. Therefore, based on our findings, we strongly recommend that community awareness about the consequences and adverse reproductive health outcomes of IPV during pregnancy should be increased. Additionally, health extension workers should be engaged in education, screening, and referral of IPV during pregnancy.

## Additional file

Additional file 1: PRISMA 2009 Checklist. (DOC 64 kb)

## Abbreviations

CI: Confidence Interval
IPV: Intimate Partner Violence
OR: Odds ratio
SNNPR: Southern Nations, Nationalities, and Peoples Region
WHO: World Health Organization
